# Moving Human Embryonic Stem Cells from Legislature to Lab: Remaining Legal and Ethical Questions

**DOI:** 10.1371/journal.pmed.0030143

**Published:** 2006-02-28

**Authors:** Henry T Greely

## Abstract

Greely discusses unanswered ethical and legal issues, such as those surrounding the creation of embryos, derivation of cell lines, uses of cell lines, and questions of intellectual property.

Research with human embryonic stem cells (hESCs) has been controversial, but in the seven years since their isolation by James Thomson [1], governments have largely answered, at least for now, the first-level questions about this research. We now know in most jurisdictions whether derivation of hESC lines will not be permitted from any embryos or whether it will be permitted from all embryos, only from embryos created as part of in vitro fertilization clinical services (IVF), or only from embryos *not* created by somatic cell nuclear transfer (SCNT). We also have learned where the various kinds of cell lines, if derived elsewhere, may be used, and whether public funding for the derivation or use of some or all of the lines will be possible. A set of second-level questions, revolving around the donors of cells and embryos used in this research, is also rapidly being answered.

I live in California, which has answered all the first-level questions with an enthusiastic endorsement of research (Box 1) and which has begun to flesh out the donor-related issues. And yet as my university gears up for hESC research, it finds that a host of other ethical and legal questions, which I will discuss in this Essay, still need to be answered.

Those questions will not be answered in a vacuum—far from it. When California researchers and their institutions receive funds from the California Institute for Regenerative Medicine (CIRM), created by Proposition 71 [2], they will be bound both by some rules set in the proposition and by other rules that CIRM will impose as regulations. Research in California not funded by CIRM is governed by standards contained in two California statutes [3,4], and by regulations that will be adopted under those statutes. Federal laws and regulations will also apply. And although technically only recommendations, the report of the National Research Council and Institute of Medicine panel on hESC research (hereafter referred to as the National Academy of Science [NAS] report) [5] will be watched closely, especially if funding agencies and journals require adherence to them. But these extensive regulations and guidelines do not come close to answering all existing questions; rather, they raise some new ones, individually and through their interaction.

The issues are discussed below in the context of California and, more broadly, the United States, but, wherever in the world this research is done (and regulated), most researchers across the globe will face most of these issues in some form (Figure 1). In many parts of the world, continuing opposition to this research means that it will be done under closer, more hostile, public scrutiny than researchers have ever experienced. It is important for hESC research and for the general standing of biomedical research with the public that researchers throughout the world answer these questions wisely.

**Figure 1:**
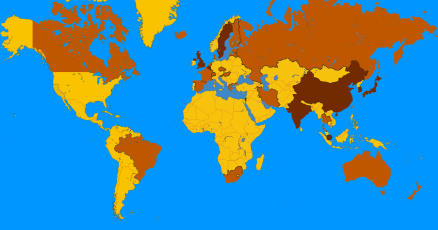
Stem Cell Policy Map Countries colored in brown have a permissive or flexible policy on human embryonic stem cell research. All have banned human reproductive cloning. These countries represent about 3.4 billion people, more than half the world's population. “Permissive” (countries in dark brown) means that various embryonic stem cell derivation techniques are permitted, including SCNT. “Flexible” (countries in light brown) means that stem cells may be derived from human embryos donated by fertility clinics only, excluding SCNT. Countries in yellow have either a restrictive policy or no established policy. (Image: William Hoffman, MBBNet [6])

The shocking fraud committed by Woo Suk Hwang and some of his group does not raise any new ethical issues—outright fabrication of results is now, and always has been, unethical. Yet Hwang's misdeeds make it even more important that hESC researchers both be fully ethical and be perceived as fully ethical in order to rebuild trust in this controversial area.

## Ethical Issues Related to Donors

All newly derived hESC lines will require embryos, eggs and sperm with which to make embryos, or eggs and somatic cells with which to make SCNT embryos. The most numerous ethical issues that must be resolved involve how the people who donate those embryos, gametes, or somatic cells should be treated. These donor-related issues have begun to receive substantial attention through laws and guidelines [3–5] and from academic commentators [7–10]. Chapter Five of the NAS report and its associated guidelines lay out what seems to be a consensus position about such donors (Box 2). Although there may be disagreement with some details of the NAS guidelines, they seem likely to be widely adopted. At least two issues relating to the donors, however, require further consideration: different kinds of donors and donor privacy.

Not all donors are ethically the same. Donors for hESC purposes may be (1) couples donating frozen embryos created for reproductive purposes, (2) couples donating “fresh” embryos created for reproductive purposes, (3) women donating fresh eggs harvested for reproductive purposes, (4) women donating fresh eggs harvested for research, (5) people donating somatic cells for use as nuclear donor cells for research, and (6) men donating sperm. The discussion of donors, including in the NAS report, has largely focused on the first category—people who might donate some of the several hundred thousand embryos that sit in American freezers. The people who had those embryos created (often, but not always, the same people who provided the eggs and sperm for their creation) will be well removed from the clinical processes of gamete donation and embryo creation, and will know more about the success of their reproductive efforts. Donors in the second, third, or fourth category—couples donating fresh embryos and women donating eggs—may raise some slightly different issues. On the other hand, because of the ease of the processes, donation of sperm and of somatic cells does not seem to raise any special problems.

Increasingly, researchers suspect that fresh embryos, not previously frozen, are more likely to lead to successful cell lines. The potential donors of these embryos will still be in the middle of their reproductive efforts and will have both less emotional distance from the decision and less knowledge of whether they might eventually want to use that embryo for themselves. For SCNT, donation of eggs will be required; egg freezing is only now emerging, in a few centers, into clinical practice. There are no freezers filled with frozen eggs that might be donated; donated eggs will come from women who have just undergone the egg donation process, a process that is at best uncomfortable and at worst life threatening—estimates are that more than 1% of women undergoing egg harvesting require hospitalization for complications. A woman who has just gone through the often difficult process of egg harvesting for reproductive uses will be asked to choose between possibly making use of potentially good eggs herself or donating them. The now-retracted 2005 *Science* paper by Hwang and his team [11] falsely claimed much greater success with eggs from younger women, who are less likely to have a clinical need for in vitro fertilization, thus raising the prospect that other researchers would also use eggs from “research donors” who are not undergoing egg harvesting for any clinical reproductive purpose. This approach may well survive Hwang's disgrace.

The differences between kinds of donors raise at least two issues. First, the differences between donating frozen embryos, on the one hand, and donating embryos and eggs newly created or harvested for reproductive purposes, on the other, may require some variation in the consent process. In the second case, the consent process and the patient's decisions will be intimately, and imminently, connected with the patient's reproductive decisions and will take place under intense time pressure. It will be important for the patient to know how many eggs or embryos, of what apparent quality, have been obtained, and to understand the clinical implications of donating eggs or embryos. Such a consent process will be harder, and will require more preparation, than consent to the donation of frozen embryos.

Second, for women undergoing egg harvest solely for research purposes, it is not clear that such donations should always be accepted. As Mildred Cho and David Magnus point out, these women would be undergoing the nontrivial risks of egg harvesting for no clinical benefit of their own, and would be more akin to living solid organ donors, whose requests to donate are often declined, than to frozen embryo donors [7]. Researchers and their institutions need to ensure, through their institutional review boards, embryonic stem cell research oversight (ESCRO) committees, or other regulatory bodies, that such donors are used only when appropriate.

The issue of privacy also needs more attention because of the long life span, and many possible uses, of hESC lines. As Bernard Lo points out, if those lines are ever to be used clinically, it not only will probably be impossible to promise donors anonymity, but it also may be necessary to warn them of continuing medical surveillance. [8] It seems highly likely that the US Food and Drug Administration will want as much information as possible about the donors' health, not only with regard to pathogens that may infect the donated cells, but also about diseases with genetic or family links. This kind of periodic recontact is itself an intrusion on donors; the fact that their identities and their whereabouts will have to be maintained by the investigators makes protection of their confidentiality more difficult. Both consequences will have to be discussed with potential donors.

## Creating Embryos

The creation of embryos for research purposes is allowed in some jurisdictions and banned in others. Even where such creation is permitted, another question may need to be answered: can nonhuman oocytes be used to create embryos through SCNT that could give rise to hESC lines? The supply of human oocytes may well turn out to be the rate-limiting factor for production of hESC lines. As early as 1998, Advanced Cell Technologies experimented with a possible way around this constraint by trying to use enucleated cow eggs as a host for a donated human nucleus [12]; in 2003, a group in Shanghai claimed to have made hESC lines using rabbit eggs. More recently, Ian Wilmut and other British scientists are seeking permission from British authorities to use rabbit eggs to create embryonic stem cells to study human disease. [13] If successful, this could substitute easily available oocytes from other mammals for hard-to-obtain human oocytes. Senator Sam Brownback has introduced legislation in the US Congress that would make the transfer of a human nucleus into a nonhuman oocyte a criminal offense [14], and such a provision has formed part of a privately announced “conservative bioethics agenda” for the second Bush Administration [15,16].

## Deriving Cell Lines

The big questions about deriving cell lines are being answered by governments, but at least one other derivation question may need to be answered: how “old” an embryo may be used to make cell lines? Most commentators have followed the 1990 British statute by setting a deadline of the appearance of the primitive streak or roughly 12–14 days of development, the time when the primitive streak can first be seen [17]. The NAS report specifically bans any “researching involving in vitro culture of any intact human embryo, regardless of derivation methods, for longer than 14 days or until formation of the primitive streak begins, whichever occurs first” [5]. It may turn out that cells can be more successfully differentiated in situ in a developing embryo than in lab equipment [18]. The justifications for using the primitive streak as a limit are more arguments of strong caution than scientifically justified lines—for example, an embryo could not have any sensation or consciousness before any neural cells differentiate, which does not occur until well after the appearance of the primitive streak. Sensation, let alone consciousness, is probably impossible for months after the formation of the primitive streak. Quick adoption of a primitive streak deadline may end up incurring major scientific costs for no ethical benefit; on the other hand, using another dividing line requires finding or justifying a bright line in what may be a smooth development process. And abandoning the primitive streak line at this point opens researchers to the claim that they will never hold to any ethical lines when it is convenient to move them.

## Using hESCs

One can imagine commercial uses for hESCs that people would find inappropriate, such as uses in cosmetics or as animal feed. At this stage in hESC research, those kinds of uses are not plausible, but one research use of hESCs has prompted substantial discussion and controversy—their use in making human/nonhuman chimeras [19–22]. Such chimeras may be useful in studying human cells and tissues in vivo in a laboratory animal, similar to the way that the SCID-hu mouse provides a model for the human immune system in an organism that can be experimented on much more freely than can humans.

The NAS panel recommended a flat ban on the creation of chimeras by inserting hESCs into the blastocysts of nonhuman primates and, out of fear that hESCs may have led to the production of human gametes, on the breeding of any nonhuman animals into which hESCs had been introduced. More broadly, it required that ESCRO committees specifically review any research that would introduce hESCs into nonhuman animals, and it advised particular caution in approving experiments putting hESCs into the brains of nonhuman animals. The Canadian Assisted Human Reproduction Act, adopted in 2004, banned the creation of chimeras defined as either the insertion of a nonhuman cell into a human embryo or an embryo with cells from more than one human embryo, fetus, or human being [23]. Senator Brownback's proposed legislation would ban several more types of chimera [14]. Researchers and institutions will have to decide whether to follow the Brownback position or the less restrictive NAS recommendations and, either way, under what circumstances ESCROs should allow the creation of permissible human/nonhuman chimeras.

## Property and Cell Lines

Researchers and their institutions will also have to deal with complicated legal, ethical, and political questions concerning property and stem cells, both intellectual property and “personal” property. The intellectual property issues for research institutions and research funders like CIRM mainly concern whether to claim, and how to assert, rights in stem cell–related discoveries; those funders need not follow the model of the federal government and its Bayh–Dole Act. The article by Merrill Goozner in *PLoS Medicine* describes many of the issues raised, including those of inexpensive access to treatments [24]. Institutions will have to decide whether to seek patents and on what terms to license them for research or other uses. This issue is especially complicated for CIRM, as the language of Proposition 71 carefully skirted this issue [2].

An equally important patent issue has received less attention. All those interested in hESC research will also have to decide how to deal with existing patents in the area. The Wisconsin Alumni Research Foundation, a nonprofit institution associated with the University of Wisconsin, owns two apparently fundamental US patents on hESCs granted to Thomson. Research institutions will have to make decisions about how to respond to these and other relevant patents by reaching an agreement with the Wisconsin Alumni Research Foundation to license them, by trying to “invent around” them [25], or possibly by litigating their validity.

Intellectual property has gotten most of the attention around stem cells, but the property in the physical cells themselves also raises issues. One of the usual consequences of ownership, the power to sell, may not apply to hESC lines. California law bans the purchase or sale of “donated cells” or of embryonic tissue, although it does allow reasonable payment for certain expenses [2,4]; the NAS guidelines contain similar recommendations. When obtaining gametes and other cells for hESC research, providing hESCs to others, and obtaining them for their own research, institutions will have to make sure that the payments involved are only for the kinds of reimbursement that fall within the statutory exceptions.

## “Border” Issues

The last set of issues that must be confronted is the most bureaucratic, the least “ethical,” and the most maddening—but not the least important. Researchers and their institutions will have to deal with many “border” issues, questions about the edges of various legal, ethical, and regulatory schemes. These include questions of time, of location, of funding, of collaboration, and of technologies.

Various hESC lines have been, and will be, created at different times. Can a researcher use a cell line that was appropriately created at the time of its creation but that would not qualify for use if it were created now? This is not a hypothetical question. The NAS guidelines, for example, require that all cell lines be derived only after permission from an ESCRO committee, but until the NAS invented them, ESCRO committees did not exist. Therefore, no cell lines derived before the NAS report, including the pre-August 2001 lines that the US government will fund, can meet that NAS recommendation. Can those cell lines be used?

Cell lines are also created in different places with different regulatory schemes. Can a cell line created legally in one country be used in another country with slightly different requirements? CIRM's proposed interim guidelines addressed this issue specifically for the United Kingdom, allowing the use of CIRM funds to do research on cell lines approved by the British Human Fertilization and Embryology Authority, but what about cell lines from South Korea, Singapore, Sweden, Israel, or Australia? And can researchers in those countries use US stem cell lines?

Funding restrictions create messier border issues. This is primarily a problem in the US for federal funds—which may not be spent to support research with hESC lines, except with those “registered lines” that comply with President Bush's policy [26]. It could also arise with other funding sources, in California and elsewhere, that limit what kinds of research can be done with their funds. Such funding limitations may sound straightforward, but they are not. What does it mean to *use* federal funds? Can an institution use a microscope owned by the federal government? What about a microscope purchased with federal funds but now owned by the institution? If a researcher's computer was purchased with federal funds, can it be used to prepare a talk about results with unregistered cell lines? If a university's E-mail system is supported in part by federal funds, can it be used for messages involving nonregistered lines? The early guidance from the federal government has been that nonconforming use of things covered by indirect-cost pools could be permitted, but they would have to be proportionately excluded from federal reimbursement. Does that mean that a university, or an individual researcher, has to track and then subtract the percentage of E-mail usage, telephone usage, electricity usage, and anything else that goes into the overhead-cost pool and involves work with unregistered cell lines?

Complying with this kind of funding restriction presents another problem. The US National Institutes of Health states clearly: “No federal funds may be used, directly or indirectly, to support research on [nonregistered] human embryonic stem cell lines…” [27]. What does it mean to use federal funds “directly or indirectly, to support research on” hESC lines? Would research with factors derived from a nonapproved cell line be able to use federal funds? Would research into genes found to be expressed in such unregistered lines be allowed or would that “support” unapproved hESC research? Can a bioethicist get federal funding to write an article about ethical problems involved in using hESC lines, or can that article be written only if it only discusses registered lines or only if it attacks (does not “support”) research with unregistered lines? All these questions depend on the definition of “research *on* human embryonic stem cell lines.”

Another important border issue involves collaborations. Assume a researcher at a California university wants to collaborate with an overseas research group. Can the researcher participate in that research only if the research meets all the requirements of US federal law, California law, the NAS guidelines, and any regulations that the university has imposed? Stem cell research is an international affair, and it would be foolish to expect each country to adopt exactly the same regulatory scheme. Generally, American university faculty cannot do human subjects research overseas without getting approval from their home institutional review board. Will such faculty have to get their home institution's ESCRO to approve—if possible—research in Singapore, Cambridge, or Seoul in which they want to collaborate? And if so, how do we define “collaborate”?

The final border issue may turn out to be the most important. The current statutes, regulations, and guidelines apply to specific “things”: embryos, embryonic stem cells, embryonic stem cell lines, etc. Each of these has its own definition, explicit or implicit. But as technology changes, hESC research may move in directions that do not fall within the existing definitions. Legislators have been wrong before; the British reproductive technologies statute banned human cloning in 1990, but the method it banned was not the one used in 1996 to clone Dolly [28]. If reprogrammed somatic cells were to make something that looked like a blastocyst, would that be an embryo giving rise to hESC lines for some, none, or all of the laws, regulations, and guidelines governing such research?

## Conclusion

These are not all the remaining ethical issues; the discussion is constrained by limits of both space and imagination. Most of the issues discussed above are not unique to hESC research, and many may seem petty, issues of detail only. But with a subject as controversial as hESC research, no detail can be considered petty. Researchers and their institutions must assume that not just their work but how they do their work will often be examined under a microscope, by hostile observers. As a result, the legal and ethical issues associated with hESC research will impose great and often unprecedented burdens on the researchers, administrators, and even lawyers for any institution that embarks on it. Coping with these issues will be painful, but not handling them well will be worse for individual researchers and institutions and for science as a whole.

**Box 1. CIRM**

In November 2004 the voters of California passed, with 59% of the vote, Proposition 71—an initiative that under California's unusual laws was placed on the ballot as a result of a petition drive by its supporters. As a result, Proposition 71 became part of California law without consideration or approval by either the legislature or the governor. (The legislature had passed laws supporting stem cell research in 2002 and 2003, but without providing any funding.) The proposition creates the new CIRM, which is governed by a 29-member “Independent Citizens Oversight Committee.” CIRM is authorized to borrow US$3 billion using California state bonds; this money is to be spent in California over ten years to support research on human stem cells, with an emphasis on types of research not fully supported by the US federal government (i.e., hESCs). The proposition has been challenged in court as violating California and federal law, and, as a result, no bonds have yet been sold, so no money has been disbursed. CIRM hopes to resolve the lawsuits soon and to begin funding research sometime in 2006. Several other American states have passed legislation supporting stem cell research, but their mechanisms are not as fleshed out as CIRM's.

**Box 2. Ethical Issues Surrounding Donors: Guidance from the NAS Report**

- All embryo, gamete, and cell donors must consent to the use of the donated material for hESC research at the time of the donation, not in advance of the donation.
- No payments, in cash or in kind, may be paid to the donors (including reimbursement or waiving of fees for storing frozen embryos), except that women who donate oocytes solely for research purposes may be reimbursed for the direct expenses of the procedure.
- Donors' decisions about their infertility treatments should be insulated from research concerns, in part by separating, whenever possible, the roles of the treating physician and the hESC researcher.
- A long list of issues must be discussed as part of the informed consent, and donors must be given the option of limiting the kinds of research that may be done with their embryos or cells, allowing, for example, derivation of hESC lines but not allowing SCNT.
- Perhaps most importantly, the NAS report requires institutions to set up ESCRO committees to oversee all aspects of this work.

## Abbreviations

CIRM: California Institute for Regenerative Medicine
ESCRO: embryonic stem cell research oversight
hESC: human embryonic stem cell
IVF: in vitro fertilization
NAS: National Acadamy of Science
SCNT: somatic cell nuclear transfer

## References

[pmed-0030143-b1] Thomson JA (1998). Embryonic stem cell lines derived from human blastocysts. Science.

[pmed-0030143-b2] The California Stem Cell Research and Cures Initiative (2004).

[pmed-0030143-b3] California Senate (2002). Stem cells: Human tissue: Research. SB 253 ch 789. http://info.sen.ca.gov/pub/01-02/bill/sen/sb_0251-0300/sb_253_bill_20020922_chaptered.pdf.

[pmed-0030143-b4] California Senate (2003). Stem cell research. SB 322 ch 506. http://info.sen.ca.gov/pub/03-04/bill/sen/sb_0301-0350/sb_322_bill_20030925_chaptered.pdf.

[pmed-0030143-b5] National Research Council and Institute of Medicine (US), Committee for Human Embryonic Stem Cell Research (2005). Guidelines for human embryonic stem cell research.

[pmed-0030143-b6] Russo E (2005). Follow the money—The politics of embryonic stem cell research. PLoS Biol.

[pmed-0030143-b7] Cho M, Magnus D (2005). Issues in oocyte donation for stem cell research. Science.

[pmed-0030143-b8] Lo B, Zettler P, Cedars MI, Gates E, Kriegstein AR (2005). A new era in the ethics of human embryonic stem cell research. Stem Cells.

[pmed-0030143-b9] Lo B, Chou V, Cedars MI, Gates E, Taylor RN (2004). Informed consent in human oocyte, embryo, and embryonic stem cell research. Fertil Steril.

[pmed-0030143-b10] Lo B, Chou V, Cedars MI, Gates E, Taylor RN (2003). Medicine. Consent from donors for embryo and stem cell research. Science.

[pmed-0030143-b11] Hwang WS, Roh SI, Lee BC, Kang SK, Kwon DK (2005). Patient-specific embryonic stem cells derived from human SCNT blastocysts. Science.

[pmed-0030143-b12] Marshall E (1998). Claim of human–cow embryo greeted with skepticism. Science.

[pmed-0030143-b13] Sample I (2006). Stem cell experts seek rabbit–human embryo. The Guardian.

[pmed-0030143-b14] US Congress (2005). Human chimera prohibition act of 2005. S. 1373, 109th Cong., 1st Sess. http://thomas.loc.gov/cgi-bin/query/z?c109:s.659.

[pmed-0030143-b15] Rick Weiss (2005). Conservatives draft a “bioethics agenda” for president.

[pmed-0030143-b16] Eric Cohen (2004). The bioethics agenda and the Bush second term. New Atlantis.

[pmed-0030143-b17] House of Commons (UK) (1990). Human Fertilization and Embryology Act of 1990, c. 37. http://www.uk-legislation.hmso.gov.uk/acts/acts1990/Ukpga_19900037_en_1.htm.

[pmed-0030143-b18] Saletan W (2005). The organ factory.

[pmed-0030143-b19] Greely HT (2003). Defining chimeras—And chimerical concerns. Am J Bioeth.

[pmed-0030143-b20] Robert JS, Baylis F (2003). Crossing species boundaries. Am J Bioeth.

[pmed-0030143-b21] Karpowicz P, Cohen CB, Van der Kooy DJ (2005). Developing human-nonhuman chimeras in human stem cell research: Ethical issues and boundaries. Kennedy Inst Ethics J.

[pmed-0030143-b22] Greene M, Schill K, Takahashi S, Bateman-House A, Beauchamp T (2005). Moral issues of human-non-human primate neural grafting. Science.

[pmed-0030143-b23] House of Commons (Canada) (2004). Assisted Human Reproduction Act, c. 2., §5(i) (the prohibition), §3 (defining chimera). http://laws.justice.gc.ca/en/a-13.4/114319.html.

[pmed-0030143-b24] Goozner M (2006). Innovation in biomedicine: Can stem cell research lead the way to affordability?. PLoS Med.

[pmed-0030143-b25] Taymor KS, Scott CT, Greely HT (2006). Stem cell technologies and stem cell-related patents. Nat Biotechnol.

[pmed-0030143-b26] Bush GW (2001). President discusses stem cell research.

[pmed-0030143-b27] National Institutes of Health (2005). Stem cell information: Frequently asked questions.

[pmed-0030143-b28] Greely HT (1998). Banning “human cloning”: A study in the difficulties of defining science. South Calif Interdiscip Law J.

